# The insoluble excretion of multi-matrix system mesalazine preparations in patients with ulcerative colitis

**DOI:** 10.1186/s12876-022-02474-9

**Published:** 2022-08-18

**Authors:** Ohtaki Yuichiro, Uchiyama Kan, Kamiya Hirotaka, Moriizumi Eri, Yamada Moe, Aoki Yuma, Watanabe Toshimune, Kiryu Sachie, Suzuki Sizuka, Matsumoto Yoshihiro, Ito Zensho, Ohkusa Toshifumi, Koido Shigeo, Saruta Masayuki

**Affiliations:** 1 Division of Gastroenterology and Hepatology, Department of Internal Medicine, The Jikei University Kashiwa Hospital, 163-1 Kashiwa, Kashiwa-shi, Chiba 277-8567 Japan; 2 Department of Microbiota Research, Juntendo University Graduate School of Medicine, 2-1-1 Hongou, Bunkyo-ku, Tokyo 113-8421 Japan; 3 Division of Gastroenterology and Hepatology, Department of Internal Medicine, The Jikei University School of Medicine, 3-19-18 Nishishinbashi, Minato-ku, Tokyo 105-0003 Japan

**Keywords:** Ulcerative Colitis, MMX, Bristol Stool Form Scale, 5-ASA, Adherence, Multi-matrix system mesalazine

## Abstract

**Background:**

Multi-matrix mesalazine (MMX) is an important treatment for ulcerative colitis (UC); however, it is often excreted intact, which increases the risk of relapse. This study aimed to clarify the risk factors for insoluble MMX excretion.

**Methods:**

The subjects were 102 UC patients who were newly prescribed MMX alone to induce remission. Their stools were evaluated on the Bristol Stool Form Scale (BSFS), the presence/absence of insoluble MMX excretion was investigated in interviews, and defecation frequency at the start of treatment and disease type were retrospectively investigated by examining their medical records.

**Results:**

The insoluble excretion rate (IER) was 14.7%. It tended to be higher in the patients with left-sided colitis or extensive colitis, although the differences among the disease types were not significant (p = 0.053). The mean defecation frequency of the patients that reported insoluble MMX excretion was significantly higher than that of the patients that did not report it (6.27 ± 5.28 vs. 3.69 ± 3.17, p < 0.05). The IER tended to be higher among the patients with soft stools (4.5%, 21.9%, and 23.1% in those with BSFS scores of ≤ 4, 5, and ≥ 6, respectively). In ROC analysis of defecation frequency, ≥ 3.5 defecations was found to exhibit sensitivity and specificity of 66.7% and 65.5%, respectively, for predicting insoluble MMX excretion.

**Conclusions:**

The likelihood of insoluble MMX excretion is influenced by defecation frequency and the extent of inflammation. It is important to keep the possibility of insoluble excretion in mind when prescribing MMX.

## Background

5-Aminosalicylic acid (5-ASA; also known as mesalazine) is able to not only induce, but also maintain, remission in many patients with active ulcerative colitis (UC), and hence, it plays a central role in the treatment of the disease. Mesalazine preparations are classified into time-dependent preparations (TDPs), in which the release of the drug depends only on time; pH-dependent preparations (PDPs), in which the release of the drug depends only on pH; and pH-dependent multi-matrix mesalazine (MMX), in which the release of the drug depends on pH and time [1, 2].

**Fig. 1:**
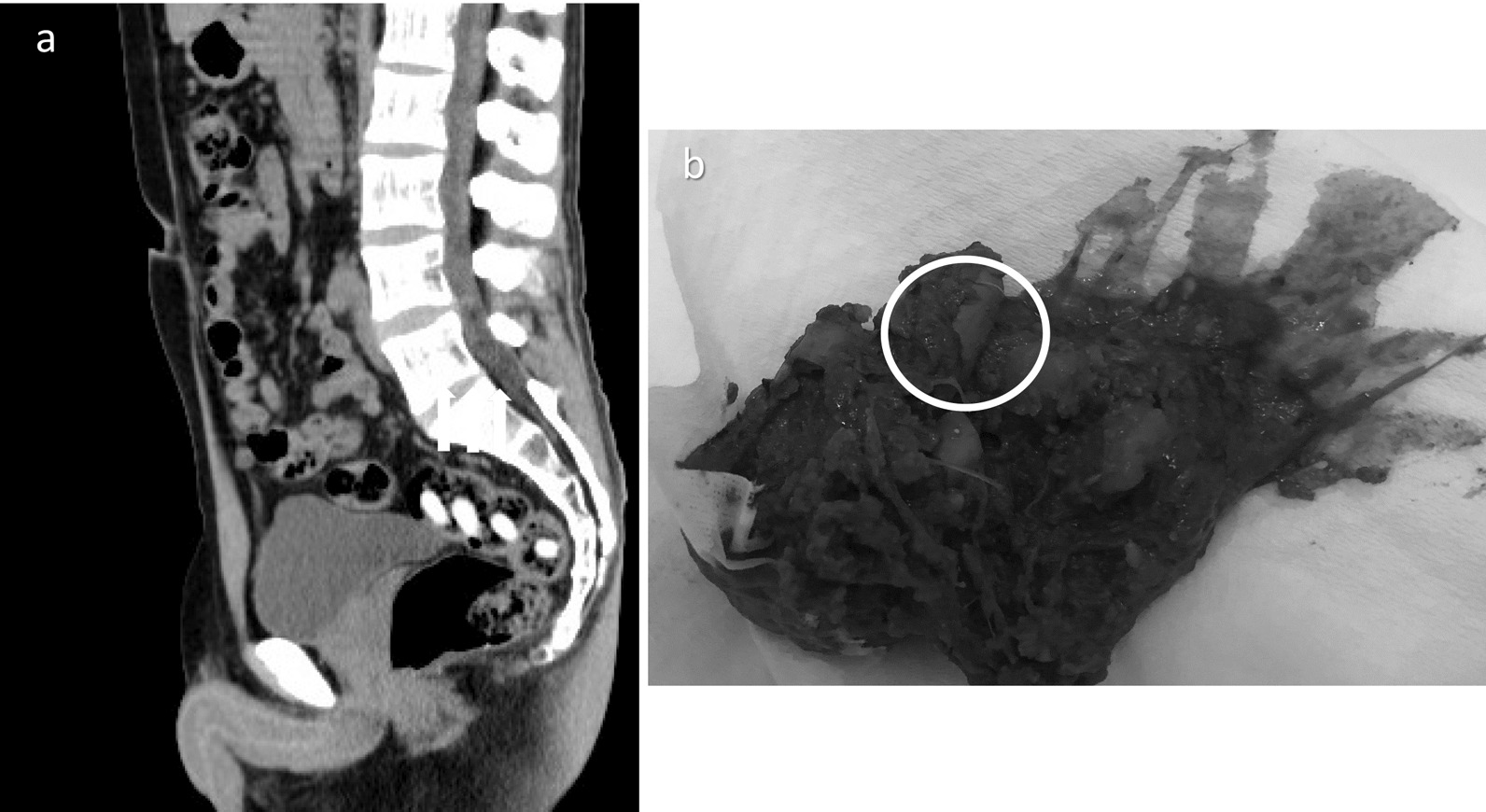
Insoluble excretion. **a** Insoluble MMX tablets in the rectum. **b** Insoluble excretion of intact tablets. Insoluble excretion was defined as the excretion of tablets with an intact pH-responsive coating, regardless of the number and frequency of excreted tablets

The main drawback of TDPs is their ability to deliver the drug to the large intestine because time-dependent formulations initiate the release of mesalazine in the small intestine. On the other hand, PDPs and MMX are expected to solve the weaknesses of TDPs. In these agents, mesalazine is encapsulated in a pH-responsive coating, which dissolves at pH 6.8–7.0. The coating is designed to dissolve near the ileocecal region and to selectively deliver mesalazine to the large intestine [3]. However, these pH-dependent agents can sometimes be seen in an undissolved state in the rectum on computed tomography, and they can even be excreted in an intact form (Fig. 1). In this study, the excretion of MMX tablets in an intact form was defined as "insoluble excretion", which increases the risk of the relapse or exacerbation of UC. The purpose of this study was to investigate the current state of insoluble MMX excretion and clarify the background factors associated with it.

## Methods

The subjects were 102 UC patients who were newly prescribed MMX and in whom remission was induced with 5-ASA alone without the use of other inducers, such as steroids, at Jikei University Kashiwa Hospital between April 1, 2016, and December 31, 2018.

**Fig. 2:**
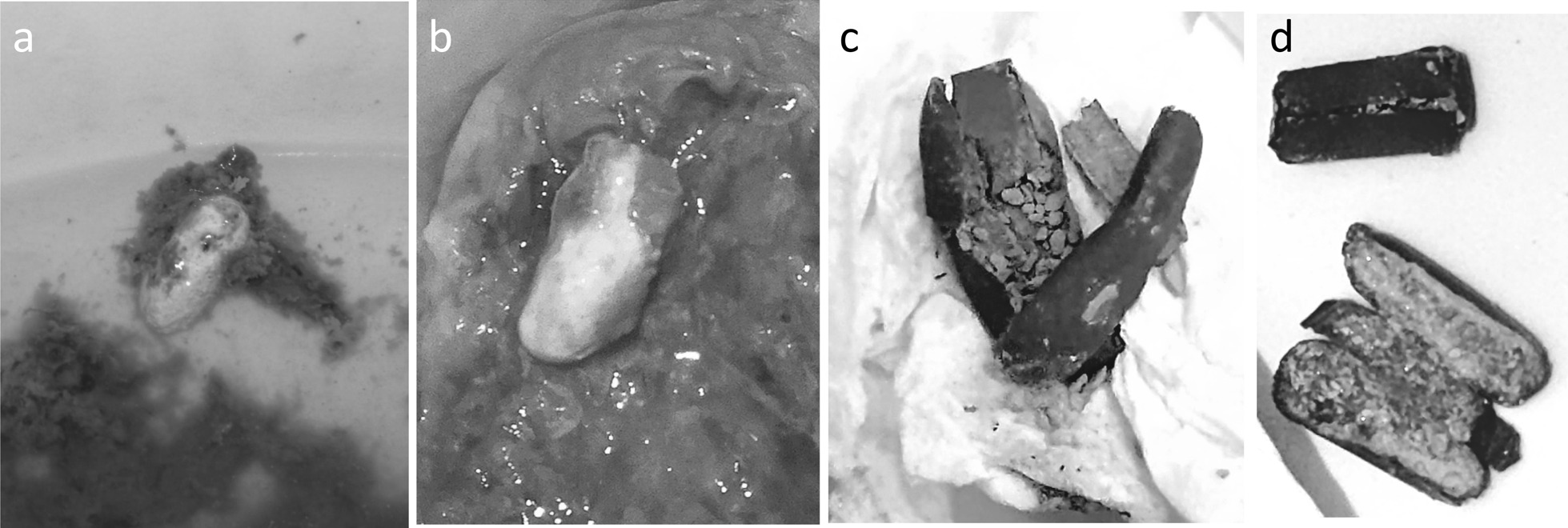
Various types of insoluble excretion. **a**, **b** “Melted tablets”, where the internal MMX is excreted with only the pH-responsive coating having dissolved. **c**, **d** So-called “ghost pills”, where the contents have disappeared, and only the coating is excreted. Cases in which excreted MMX had been exposed due to part of the coating having dissolved, and those involving so-called "ghost pills", where the contents of the tablet had disappeared, and only the coating was excreted, were not counted as insoluble excretion in this study

We retrospectively investigated defecation frequency at the start of treatment, stool consistency, disease type, and the presence or absence of insoluble excretion. Defecation frequency was assessed based on the number of times that stools were actually discharged, rather than the number of times patients went to the toilet with an urge to defecate. Patients that were prescribed pH-dependent MMX preparations were asked about insoluble excretion at each visit (every 1–3 months) for 1 year after the drug was first prescribed. In this study, we investigated whether these patients experienced insoluble excretion. We defined insoluble excretion as the excretion of MMX tablets with an intact pH-responsive coating; i.e., in an undissolved state, regardless of the number and frequency of excreted tablets. Cases in which the MMX was exposed due to part of the coating having dissolved, and those involving so-called "ghost pills"; i.e., when the contents of the pills had disappeared, and only the coating was excreted, were not counted as insoluble excretion (Fig. 2).

All patients underwent lower colonoscopy within the year prior to the start of MMX treatment to assess their disease activity level and the type of UC that they had. Inflammation that extended beyond the splenic flexure, up to the splenic flexure, and up to the sigmoid colon was defined as extensive colitis, left-sided colitis, and proctosigmoiditis, respectively, whereas inflammation that was restricted to the rectum alone was defined as proctitis.

The Bristol Stool Form Scale (BSFS) was used to assess the consistency of the patients’ stools, and the patients were classified into three groups: BSFS score: ≤ 4, solid stools; BSFS score = 5, tangibly soft stools; and BSFS score: ≥ 6, muddy stools and diarrhea [4, 5].

All statistical analyses were performed using IBM SPSS Statistics, version 22. P-values of < 0.05 were considered to indicate statistical significance.

## Results

MMX preparations were prescribed to 102 patients. Table 1 shows for the patient background data. The mean age of the patients was 41.6 ± 14.7 years, and there were 49 males and 53 females. At the start of treatment, 66, 35, and 1 patient(s) exhibited mild, moderate, and severe disease, respectively. The disease types were proctitis in 13 patients, proctosigmoiditis in 18 patients, left-sided colitis in 10 patients, and extensive colitis in 61 patients. The mean defecation frequency at the start of treatment was 3.91 ± 3.29 times/day. The BSFS score at the start of treatment was ≤ 4 in 44 patients, 5 in 32 patients, and ≥ 6 in 26 patients (Table 1).

**Table 1:** Characteristics

	n = 102
Age	41.6 ± 14.7
Sex (male: female)	49: 53
Inflammatory findings (extensive colitis: left-sided colitis: proctosigmoiditis: proctitis)	13: 18: 10: 61
Defecation frequency at the start of treatment	3.91 ± 3.29
Bristol Stool Form Scale score at the start of treatment (≤ 4: 5: ≥ 6)	44: 32: 26
UC disease activity at the start of treatment (mild: moderate: severe)	66: 35: 1
Concomitant drugs (none: proton pump inhibitors: H2 receptor antagonists)	74: 9: 19

**Fig. 3:**
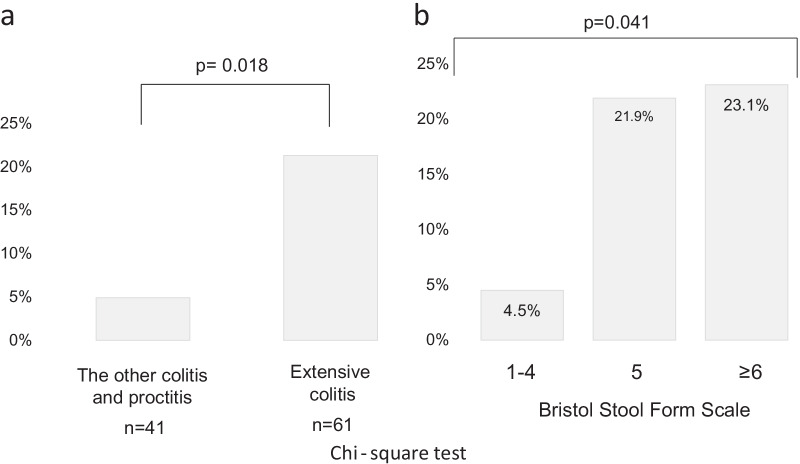
Insoluble excretion rate according to the extent of inflammation and stool consistency

The insoluble excretion rate of MMX was 14.7% (15 out of 102 patients). In addition, the insoluble excretion rate was 0% in the proctitis group, 0% in the proctosigmoiditis group, 20.0% (2/10) in the left-sided colitis group, and 21.3% (13/61) in the extensive colitis group. Although the insoluble excretion rate tended to be higher in the left-sided colitis and extensive colitis groups, the differences among the disease types were not statistically significant (p = 0.053). However, when the disease types were classified into two groups, the insoluble excretion rate was significantly higher in the extensive colitis group than in the other groups (21.3% vs. 4.9%, p = 0.018) (Fig. 3a). The insoluble excretion rate was 4.5% (2/44 patients), 21.9% (7/32 patients), and 23.1% (6/26 patients) in the patients with BSFS scores of ≤ 4, 5, and ≥ 6, respectively (Fig. 3b).

**Fig. 4:**
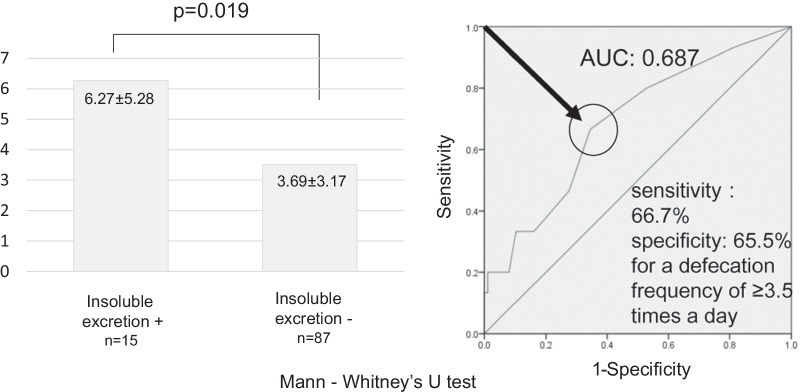
A comparison of defecation frequency and ROC curve analysis. **a** A comparison of defecation frequency at the start of MMX treatment. **b** ROC curve analysis of defecation frequency and insoluble excretion. ROC analysis in which the defecation frequency at the start of treatment was employed as the test variable indicted that a defecation frequency of ≥ 3.5 times a day exhibited sensitivity and specificity of 66.7% and 65.5%, respectively, for predicting insoluble MMX excretion

When the mean defecation frequency at the start of MMX treatment was compared between the patients that did and did not experience insoluble excretion, it was found that the mean defecation frequency of the patients that experienced insoluble excretion was significantly higher than that of the patients that did not experience it (6.27 ± 5.28 times/day vs. 3.69 ± 3.17 times/day, p < 0.05) (Fig. 4a).

ROC analysis in which the defecation frequency at the start of treatment was employed as the test variable indicated that a defecation frequency of ≥ 3.5 times a day exhibited sensitivity and specificity of 66.7% and 65.5%, respectively, for predicting the insoluble excretion of MMX (Fig. 4b).

Concomitant use of proton pump inhibitors (PPIs) and H2 receptor antagonists (H2RAs) is considered to affect the lysis of pH-dependent 5-ASA preparations [6–8]. In this study, the insoluble excretion rate was 6% lower in the patients who used PPIs or H2RAs in combination with 5-ASA. However, there were no significant differences in the insoluble excretion rate between the patients taking 5-ASA alone, PPIs with 5-ASA, and H2RAs with 5-ASA.

## Discussion

5-ASA is a positional isomer of para-aminosalicylic acid (4-ASA), an antibacterial drug used to treat rheumatoid arthritis. It has been used since the 1940s, making it the longest-established drug for the treatment of inflammatory bowel disease [9]. The development of 5-ASA preparations began with salazosulfapyridine. Salazosulfapyridine is an azo compound consisting of mesalazine and sulfapyridine and has a unique drug delivery system (DDS), which selectively releases mesalazine into the colon via the cleavage of the azo bond by azoreductase, a bacterial enzyme found in the colon [10]. Due to its high efficacy, it is still used in the treatment of UC. However, the side effects of salazosulfapyridine, such as the pigmentation of sweat, tears, and urine and skin rashes, are considered to be problematic, and sulfapyridine has been suggested to contribute to these effects. Mesalazine-only drugs have been developed to avoid the various side effects of salazosulfapyridine. They include TDPs coated with a porous film of mesalazine and ethylcellulose and PDPs coated with a film of methacrylic acid copolymer [11, 12]. TDPs have the disadvantage that the mesalazine begins to be released from the upper jejunum, and it rarely reaches the distal colon, even when it is administered at a dose of 4.0 g/day, the maximum dose approved by the Japanese national health insurance system. PDPs, on the other hand, have a DDS in which the coating dissolves near the end of the ileum at pH ≥ 6.8 to 7.0, and hence, more mesalazine is delivered to the distal colon [11, 12]. PDPs are approved for use in Japan at doses up to 3.6 g/day, but even at a dose of 2.4 g/day, the intramucosal mesalazine concentration in the sigmoid colon is significantly higher than that achieved by TDPs at a dose of 3 g/day [13]. Furthermore, pH- and time-dependent MMX formulations consist of a tablet, containing mesalazine dispersed in a matrix consisting of a hydrophilic and lipophilic base, coated with a pH-responsive polymer film [14]. When the drug is transported to the large intestine, the polymer film dissolves, the tablet is exposed to intestinal fluid, and the hydrophilic and lipophilic base inhibit the penetration of intestinal fluid into the tablet, resulting in the gradual release of mesalazine into the large intestine. MMX preparations have been approved for use at a maximum dose of 4.8 g/day under the Japanese national health insurance system. They are reported to produce high mesalazine concentrations in the distal large intestine mucosa, although the concentrations achieved are not significantly different from those achieved by TDPs and PDPs [15]. Furthermore, MMX is administered once a day from the active period to the remission period, and hence, its use is expected to improve medication adherence [2, 16].

In this way, various measures have been taken to improve the deliverability of mesalazine to the large intestine. In fact, it has been reported that the intramucosal mesalazine concentration in the distal colon increases in the following order: TDPs, PDPs, and MMX [15]. Although PDPs and MMX were developed to efficiently release mesalazine into the colon, we often encounter cases of unwanted elimination of such tablets in patients with worsening UC and an increased frequency of defecation. Due to the release mechanism of MMX tablets, if the coating of such tablets is intact when the tablets are excreted (insoluble excretion) the mesalazine within the tablets will not have been released, which may increase the risk of relapse or exacerbation. In fact, in this study a high defecation frequency at the start of treatment was found to be a risk factor for the insoluble excretion of MMX, regardless of the type of colitis present.

In a previous study, we examined unwanted excretion in 95 patients who were newly started on PDPs at our hospital between 2014 and 2018, and the insoluble excretion rate was 12.6% (12/95 cases) (data not shown). There was no significant difference in the insoluble excretion rate between different types of disease: 2% (3/18 cases) for proctitis, 4% (6/15 cases) for proctosigmoiditis, 0% (0/11 cases) for left-sided colitis, and 6% (3/51 cases) for extensive colitis. As for the effects of stool consistency at the start of treatment, 33.3% (7/22 cases) of the patients with a BSFS score of ≥ 6, 16.7% (2/12 cases) of those with a BSFS score of 5, and 8.3% (2/28 cases) of those with a BSFS score of ≤ 4 experienced insoluble excretion, as did 9% (3/33 cases) of the patients whose defecation frequency was unknown. However, there was no significant difference in the frequency of insoluble excretion among the groups, suggesting that the insoluble excretion of PDPs is less predictable than that of MMX.

In an in vitro study, Abinusawa A et al. compared the rate of mesalazine release from various 5-ASA formulations in acidic environments that changed over time from pH 1 to pH 6.0 and pH 6.8 [17]. As a result, it was found that TDPs release mesalazine at a constant rate over time, completely independent of pH, while PDPs and MMX only begin to release mesalazine at pH 6.8, indicating that the rate of release differs greatly between PDPs and MMX. In other words, in the case of PDPs almost 100% of the mesalazine is released within 2 h after the environmental pH reaches 6.8, whereas for MMX it takes approximately 6 to 7 h for 100% of the mesalazine to be released because the encapsulated tablet is protected by the film coating, and the mesalazine is only released after the coating dissolves.

The stagnation time of colonic contents is strongly influenced by the frequency of defecation. In other words, except for special cases, such as patients with tenesmus, more frequent defecation shortens the stagnation time in the large intestine, which is considered to be the reason why the insoluble excretion rate of MMX increases with the number of defecations. The frequency of defecation and fecal characteristics during the active phase of the disease are strongly influenced by the extent of the disease, suggesting that the disease type is a risk factor for insoluble MMX excretion.

In the photographs shown in Fig. 2, a clear difference in the dissolution process of the pH-responsive film coating can be seen between PDPs and MMX. In the case of MMX, the surface of the coating dissolves first, whereas in the case of PDPs the edges dissolve first. It is considered that the edges of PDPs dissolve faster than the surface, and the undissolved surface peels away easily (Fig. 2c), with the mesalazine inside being released within a short period of time. In fact, during lower endoscopy, we often encounter PDPs as so-called ghost pills; i.e., pills from which the mesalazine has been released into the colon, leaving only the outer shell. Therefore, PDPs are considered to be affected less by defecation frequency than MMX.

One of the consequences of insoluble excretion is that the remission-inducing or remission-maintaining ability of the drug in question cannot be fully elucidated. However, the insoluble excretion of a portion of a dose may have little effect on a patient’s clinical course, depending on the frequency of such excretion, the extent of their lesions, and the degree of inflammation. We considered that it would not be possible to determine whether insoluble excretion itself increased disease activity in this study, and hence, this matter was not investigated.

Both physicians and patients are generally unaware of insoluble excretion occurring; therefore, there is a risk that additional treatments, such as steroids, immunomodulators, or biologics, may be added unnecessarily. In many of these cases, it may be possible to induce remission by optimizing the mesalazine preparation, e.g., by switching it. In fact, it has been reported that mesalazine switching is effective in 33–59% of cases [18]. Adding the abovementioned treatments to such cases would represent a form of medical malpractice and would also have a considerable impact on medical costs. Therefore, when PDPs or MMX are selected, it is necessary to explain the possibility of insoluble excretion to patients in advance and have them monitor their stools.

This study had several limitations. First, it was a single-center retrospective study involving a small number of cases. In addition, tablets can be easily buried in solid stools, making them difficult to see, whereas they may be easier to see in muddy stools and diarrhea. Also, it is considered that patients are more interested in their feces during the active phase of UC, and as a result they may notice insoluble excretion more often during this period. Conversely, patients in remission, who have less interest in their excrement, may not be aware of insoluble excretion. Another important limitation of this study was the visual method used to detect insoluble excretion, the effectiveness of which may have been affected by the amount of fecal material produced and/or the absolute amount of insoluble excretion. Therefore, there may have been cases in which insoluble excretion was overlooked.

In this study, insoluble excretion was defined as when the pH-responsive coating of tablets did not dissolve, and the tablets were excreted in their original form. Due to the characteristics of MMX formulations, it is difficult for so-called “ghost pills” (Figs. 2c,d), in which the outer shell partially dissolves and the mesalazine inside is released, to form. On the other hand, there are many cases in which the outer shell dissolves, but the MMX inside is excreted as a “melted tablet” (Figs. 2a,b). Thus, we have started to measure urinary salicylic acid levels to detect whether the internal drug has been assimilated, and a prospective study including such measurements should be performed [19, 20].

In order to clarify the drug selection criteria for UC, the definition of insoluble excretion needs to be reexamined, and a prospective study is required to prevent cases of insoluble excretion from being overlooked. It is also necessary to assess whether insoluble excretion exacerbates UC or whether insoluble excretion occurs due to an increase in the defecation frequency associated with the exacerbation of UC. It is hoped that further research will make it possible to establish more accurate drug usage standards for UC.

In conclusion, the likelihood of insoluble MMX excretion is influenced by defecation frequency and the extent of inflammation. However, clinicians should not avoid prescribing MMX based on the patient’s defecation frequency or disease activity. Rather, it is important that both physicians and patients are aware that insoluble excretion of MMX can occur, as it can delay healing.

## Data Availability

All relevant data are included within the manuscript.
