# Correction: The insoluble excretion of multi-matrix system mesalazine preparations in patients with ulcerative colitis

**DOI:** 10.1186/s12876-022-02608-z

**Published:** 2023-02-02

**Authors:** Yuichiro Ohtaki, Kan Uchiyama, Hirotaka Kamiya, Eri Moriizumi, Moe Yamada, Yuma Aoki, Toshimune Watanabe, Sachie Kiryu, Shizuka Suzuki, Yoshihiro Matsumoto, Zensho Ito, Toshifumi Ohkusa, Shigeo Koido, Masayuki Saruta

**Affiliations:** 1 Division of Gastroenterology and Hepatology, Department of Internal Medicine, The Jikei University Kashiwa Hospital, 163-1 Kashiwa, Kashiwa-shi, Chiba 277-8567 Japan; 2 Department of Microbiota Research, Juntendo University Graduate School of Medicine, 2-1-1 Hongou, Bunkyo-ku, Tokyo 113-8421 Japan; 3 Division of Gastroenterology and Hepatology, Department of Internal Medicine, The Jikei University School of Medicine, 3-19-18 Nishishinbashi, Minato-ku, Tokyo 105-0003 Japan

**Correction: BMC Gastroenterology (2022) 22:390** https://doi.org/10.1186/s12876-022-02474-9

After publication of this article [1], the authors reported that the given and family names of all authors were incorrectly structured.

The original article [1] has been corrected.
